# Toward compassionate workplaces: Employers’ and employees’ perspectives on serious illness, dying, death, and grief: Findings from a nationwide online survey in Germany

**DOI:** 10.1371/journal.pone.0345333

**Published:** 2026-04-17

**Authors:** Helena Kukla, Tobias Lang, Melanie Joshi, Karin Ohler, Golrokh Esmaili, Georg Bollig, Raymond Voltz, Julia Strupp

**Affiliations:** 1 Department of Palliative Medicine, Faculty of Medicine and University Hospital Cologne, University of Cologne, Cologne, Germany; 2 Department of Anesthesiology, Intensive Care, Palliative Medicine and Pain Therapy, HELIOS Klinikum, Schleswig, Germany; 3 Center for Health Services Research, Faculty of Medicine and University Hospital Cologne, University of Cologne, Cologne, Germany; 4 Center for Integrated Oncology Aachen Bonn Cologne Dusseldorf (CIO ABCD), Faculty of Medicine and University Hospital Cologne, University of Cologne, Cologne, Germany; The Hong Kong Polytechnic University, HONG KONG

## Abstract

**Background:**

Experiences of serious illness, dying, death, and grief in the workplace significantly affect employee well-being, productivity, and organizational culture. Despite increasing recognition of their importance, there is limited understanding of workplace perspectives in Germany. This study addresses this gap by examining the attitudes and experiences of employees and employers regarding these issues to inform workplace policies that promote well-being and resilience.

**Methods:**

We conducted a nationwide representative cross-sectional online survey (n = 1,127) using a self-developed questionnaire with closed and open-ended items. Data were collected from managers and non-managers across multiple sectors to identify key themes, challenges, and opportunities for improving workplace practices. Closed-ended questions were analyzed quantitatively, and open-ended responses qualitatively.

**Findings:**

More than sixty-three percent (63.1%) of respondents reported having been affected within the past five years. Workplace support was limited, with 42.7% seldom or never receiving managerial support. Informal support from colleagues and managers was more common and often preferred over formal provisions. Employees valued understanding, empathy, and availability, whereas managers emphasized structural measures such as leave policies. Barriers, uncertainties, and a strong need for guidance and training were frequently reported. Employees in smaller companies expressed greater concerns about legal consequences and stronger support needs, while larger organizations more often offered formal guidelines and programs.

**Conclusions:**

The mismatch between employees’ needs and managerial focus underscores the importance of aligning workplace practices with employee expectations. Training, guidance, and initiatives that integrate psychosocial and structural support could strengthen well-being and enhance organizational resilience.

## Introduction

Experiences of serious illness, dying, death, and grief in the workplace are significant yet often overlooked aspects of organizational life. Research indicates that these issues have a profound effect on workplace dynamics and employee well-being [1].

In Germany, a total of 1,002,741 people died in 2024, of whom approximately 281,504 (28%) were of working age (15–67 years) [2]. It is reasonable to assume that every working individual will encounter issues relating to serious illness, dying, death, or grief in the workplace at some point in their career [3]. As the population ages, the prevalence of bereavement at work is expected to increase, especially due to the aging parents of the baby boomer generation, leading to more frequent encounters with grieving colleagues [4]. Wilson [5] refers to this trend as a looming “tsunami of grief.”

Workplace grief arises not only from the death of colleagues, but also as anticipatory grief resulting from serious illness of loved ones in the employees’ personal lives [6]. Caring for sick relatives is becoming increasingly common and often must be managed alongside work and childcare obligations. In a previous study conducted by our team (Kasdorf et al., 2024), 28% of respondents reported having taken sick leave to care for a relative. Similarly, Pieper et al. [7] highlighted that employees expressed clear needs and expectations for workplace support to better reconcile work and caregiving responsibilities, including greater flexibility in working hours. This “sandwich” position contributes to physical, emotional, and financial stress, and may lead to neglect of personal health [8]. The impact of grief extends beyond the individual, affecting colleagues, workplace dynamics, and overall productivity. It can cause significant mental health strain, increased absenteeism, reduced motivation, and impaired concentration [9–12].

While the workplace can provide stability and distraction during grief, it can also undermine well-being when colleagues respond unsympathetically [13]. Effective support requires balancing realistic work expectations with empathy and emotional intelligence. However, a lack of clear guidelines and limited experience often creates uncertainty when it comes to addressing grief in the workplace [14,15]. To better support grieving employees, organizations should implement clear policies, flexible arrangements, and proactive support measures [16].

Challenges remain in navigating support systems and addressing disenfranchised grief, particularly in high-pressure work environments [15]. Managers play a pivotal role in this process, especially when equipped with the appropriate policies and training [11].

To date, research on supportive work environments and evidence-based interventions for employees experiencing serious illness, dying, death, and grief remains limited [16]. The present study is part of the project “Last Aid in the Workplace as a sensitive approach to death and grief” (LAUT) and aims to fill this gap by documenting experiences of managerial and colleague behaviors in German companies regarding serious illness, dying, death, and grief and existing strategies. The findings will inform the development of a comprehensive framework to promote a more sensitive and supportive workplace culture around serious illness, dying, death, and grief.

## Materials and methods

A nationwide online survey was conducted in Germany between April 2024 and May 2024. This study received IRB approval from the Medical Ethics Review Board of the Medical Faculty at the University of Cologne (23–1443_1) and was registered with the German Register for Clinical Trials (DRKS00033422). Survey responses did not contain any personally identifiable information. Reporting of the study adheres to the CHERRIES guideline for online survey studies [17].

### Survey development and design

Due to the lack of a standardized questionnaire addressing issues of serious illness, dying, death, and grief in the workplace, a project intern group (HK, JS, and TL) developed a cross-sectional survey instrument inductively based on a focused literature review. Relevant aspects and preliminary questions were first generated through brainstorming. The multidisciplinary research team—comprising experts in health sciences, psychology, palliative medicine, and sociology—then refined the survey items through iterative discussion and consensus-building.

The questionnaire was designed as an exploratory instrument addressing multiple heterogeneous domains (e.g., experiences, attitudes, organizational practices, and support needs), rather than as a unidimensional scale. Consequently, internal consistency measures such as Cronbach’s alpha or factor analysis were not calculated.

This version of the survey included 34 closed questions, two semi-open questions and six open-ended questions, and took approximately 30 minutes to complete in order to collect comprehensive, in-depth data. Managers with personnel responsibilities received an additional item on possible supportive actions for affected employees. The closed-ended questions used Likert scales and multiple-choice response options to obtain a comprehensive assessment of workplace issues. The open-ended questions aimed to capture subjective experiences, narratives, practices and aspirations for change.

The initial version of the survey was pre-tested with representatives of the target population to ensure its validity and comprehensiveness using the ‘think aloud’ method [18]. The pre-testing process involved iterative cycles of feedback and revision until the survey items worked as intended. The questionnaire was finalized after pre-testing with seven participants, focusing primarily on improving the clarity of item wording and coherence of content.

Participants were given the opportunity to revise their responses using a back button during the completion of the questionnaire.

The final survey covered the following topics:

1. Demographic data
2. Organizational structures related to serious illness, dying, death, and grief
3. Personal experiences with serious illness, dying, death, and grief in the workplace
4. Strategies for supporting affected colleagues
5. Wishes and needs for improving approaches to serious illness, dying, death, and grief in the workplace

### Sampling and recruitment

Participants were eligible if:

1. they were 18 years or older;
2. they were employed;
3. they had sufficient literacy skills to complete a questionnaire in German;
4. they gave electronic informed consent.

A non-probability quota sampling method was used to recruit a representative sample of employed people in Germany in terms of age and federal state.

Our target sample size was *n* = 1,000. We exceeded the original target, reaching a size of 1,127 which further increased statistical power [19]. Recruitment was facilitated by uzbonn GmbH (uzbonn.de), an external partner specializing in empirical social research and evaluation, who programmed the questionnaire, hosted the panel survey and distributed the invitation emails. Uzbonn GmbH collaborates with Bildendi GmbH, a professional market research company specializing in the recruitment of online survey panels.

The panel provider maintained a pool of registered members who had previously consented to participate in surveys. The invitation included detailed information about the study’s purpose and estimated completion time. Interested individuals were required to complete a registration process, during which they provided personal information that was used for participant segmentation and selection. Once the target sample size had been reached, the selected participants were notified via email that the survey was about to begin. In addition, a combination of social media outreach and snowball sampling was used. Participants provided electronic informed consent and agreed to anonymous data processing; no written consent was required due to the minimal-risk and non-clinical nature of the study. Participants were categorized into three groups to analyze potential differences: managers with personnel responsibility, managers without personnel responsibility, and non-managerial employees. All participants received monetary compensation for their participation.

### Data collection and analysis

The data were collected between 8 April 2024 and 22 April 2024. Closed items were analyzed descriptively using SPSS (Version 29). Pseudo-*R*² measures (Cox & Snell, Nagelkerke, McFadden) were used to assess model fit. For nominal-to-interval relationships (company size × presence of guidelines, initiatives, or contact points), Eta² was reported to quantify effect sizes.

All free-text responses in the final dataset were imported into MAXQDA Analytics Pro 2024 [20] for thematic analysis using the methodology outlined by Braun and Clarke [21]. All qualitative responses were originally collected in German. For publication purposes, illustrative quotations were translated into English by members of the research team with expertise in both languages, with careful attention to preserving meaning and contextual nuance. This inductive methodology ensures that emerging codes (individual concepts associated with specific data segments) and themes (patterns of shared meaning) are grounded in the original dataset. Using the six-phase model, the transcripts were independently analyzed as follows:

1. HK (a health scientist) and TL (a psychologist), both with extensive expertise in qualitative data analysis, independently familiarized themselves with the data.
2. Both researchers independently carried out line-by-line coding of one third of the material.
3. They discussed and reconciled the similarities and discrepancies in their coding, and independently organized them into themes until a consensual scheme of themes and codes was established.
4. Using this scheme, HK analyzed half of the material, while TL reviewed and refined the themes and associated codes.
5. The differences identified by the two authors were integrated to produce a final version of the codebook.
6. TL then carried out comprehensive coding of all responses based on the established codebook. Ambiguous segments were reviewed collaboratively by HK and TL until consensus was reached.

Due to ethical and legal restrictions, the qualitative free-text comments and quantitative survey data cannot be shared outside the project team; data are stored securely and access can be granted upon reasonable request to the corresponding author or the Department of Palliative Medicine, University Hospital Cologne.

## Results

### Sample

Invitations to participate in the survey were sent to 15,203 individuals via uzbonn. Of those invited, 15% (*n* = 2,280) started the survey by completing the informed consent form and the first page of the questionnaire. The completion rate, defined as the proportion of participants who submitted the final page of the questionnaire, was 47% (*n* = 1,068 out of 2,280), corresponding to 7% of all invitees. Additionally, 59 participants were recruited via snowballing (*n* = 1,127 in total). The final sample was representative of the German working population with respect to age, federal state, and position (manager vs. non-manager). The mean age was 43 with a range of 18–64 years. The distribution of respondents by age, gender, company size, and current position is shown in Table 1.

**Table 1:** Distribution of Respondents by Age, Gender, Company Size, and Current Position.

Variable	Category / Class	n	%
**Age**	18–24 years	123	10.9
	25–34 years	223	19.8
	35–44 years	226	20.1
	45–54 years	286	25.4
	55–64 years	269	23.9
**Gender**	Female	589	52.3
	Male	535	47.5
	Diverse	3	0.3
**Company size**	1–10 employees	137	12.2
	11–50 employees	227	20.1
	51–250 employees	254	22.5
	>250 employees	509	45.2
**Current position**	Manager with personnel responsibility	369	32.7
	Manager without personnel responsibility	204	18.1
	Non-manager	554	49.2

**Note.** *n* = absolute frequency; % = proportion of total sample. Percentages may not sum to 100% due to rounding.

### Impact of critical life events

The majority of respondents (85.2%) had observed a colleague or manager taking the standard two days of paid bereavement leave or sick leave due to serious illness, dying, death, or grief, 63.1% of respondents who completed this item reported having experienced serious illness, dying, death, or grief within the last five years.

### Personal experiences and their impact in the workplace

In our cohort, 14.8% reported that end-of-life and bereavement issues affected their work-related activities every day or almost every day, while 29.1% on several days and 45.8% reported an impact on some days.

Of those affected by the event, 26.8% reported being unable to carry out their work as usual, while 34.2% said they were able to maintain their usual work performance, and 39.0% reported doing so only partially.

### Dealing with serious illness, dying, death, and grief

15.5% of respondents stated that they had never discussed their own concerns at work with their managers and/or co-workers, while 14.1% said that they had often or very often discussed how they felt affected. 16.8% have often or very often felt uncomfortable at work, whereas 23% have never felt uncomfortable at work due to their emotional state. 44.6% have tried to suppress their emotions at work, compared to 13.7% who said they have never done so.

### Support from colleagues and managers

Just over one quarter of participants (28.6%) reported often or very often receiving support. By contrast, 19.3% indicated that they had never received such support, and 23.4% stated that they rarely did. Support from colleagues was reported as often or very often by 40.4% of participants, while 11.2% indicated that they never received support, 15.7% reported receiving support rarely, and 32.7% sometimes.

Additionally, 53.4% of participants indicated that they sometimes, often, or very often wished for more support from their employer regarding changes in working conditions (e.g., remote work, reduced working hours, flexible scheduling). Conversely, 24.0% stated that they never desired more support, and 21.6% stated they seldom did. Regarding emotional support, 41.2% of participants sometimes, often, or very often wished for more support from their supervisor or colleagues, while 24.8% never wished for more support and 26.0% wished for more support rarely.

### Effects of company size on workplace support and concerns

Nearly three quarters of respondents (74.7%) reported that there were no initiatives to support employees dealing with serious illness, dying, death, and grief in the workplace. Moreover, 66.8% did not have access to a designated contact points for such matters.

Ordinal logistic regression analyses indicated that company size explained only a small proportion of the variance in most outcomes (Pseudo *R*² ≤ 0.025). Employees and employers in companies with 11–50 employees were significantly more likely to report concern about legal consequences (*β* = 0.50, *OR* = 1.65, *p* = .007) and had a stronger desire for workplace support regarding both working conditions (*β* = 0.49, *OR*=1.64, *p* = .006) and emotional support (*β* = 0.45, *OR*=1.56, *p* = .012) than the reference group (>250 employees). Similar trends were observed for companies with 51–250 employees, particularly for support-related outcomes. Other outcomes, such as perceived empathy or support from colleagues/supervisors, generally did not differ significantly by company size.

Regarding institutional support, the presence of workplace guidelines, initiatives/programs and dedicated contact points was positively associated with company size (eta² = 0.033, 0.066, and 0.096, respectively; all *p* < .001), indicating that larger companies were more likely to provide structured support for employees affected by serious illness, dying, death, or grief. Table 2 presents the regression results and company support measures by company size.

**Table 2:** Regression Results and Company Support by Company Size.

Variable / Measure	Company Size	β	p	OR / Eta²	Significance
**Concern about legal consequences at work**	1–10 employees	0.217	0.333	1.24	n.s.
	11–50 employees	0.500	0.007	1.65	p < 0.01
	51–250 employees	0.299	0.108	1.35	n.s.
	>250 employees	0	–	1	Reference
**Desire for support regarding working conditions**	1–10 employees	−0.279	0.187	0.76	n.s.
	11–50 employees	0.493	0.006	1.64	p < 0.01
	51–250 employees	0.406	0.021	1.50	p < 0.05
	>250 employees	0	–	1	Reference
**Desire for emotional support**	1–10 employees	0.433	0.042	0.65	p < 0.05
	11–50 employees	0.446	0.012	1.56	p < 0.05
	51–250 employees	0.355	0.043	1.43	p < 0.05
	>250 employees	0	–	1	Reference
**Company support – Guidelines available**	–	–	–	Eta² = 0.033	p < 0.001
**Company support – Initiatives / programs available**	–	–	–	Eta² = 0.066	p < 0.001
**Company support – Contact points available**	–	–	–	Eta² = 0.096	p < 0.001

**Note.** β = standardized regression coefficient; OR = odds ratio; Eta² = effect size; n.s. = not significant. Reference category for company size: > 250 employees. Beta values and OR rounded to three decimal places.

## Free-text responses: Experiences with serious illness, dying, death, and grief in the workplace

### Existence of workplace initiatives or programs

Of the 1,127 participants, 256 (23%) reported the existence of initiatives or programs designed to support individuals dealing with serious illness, dying, death, or grief in the workplace. Structural offerings (*n* = 90 codes), such as time off work or financial support, predominate compared to psychosocial offerings (*n* = 60 codes), such as personal conversations, or grief counselling by psychologists, pastoral counsellors, social workers, supervisors or colleagues. Only 12 respondents reported training or educational offers.

### Experienced forms of support from colleagues, staff, or supervisors

A total of 327 participants responded to this semi-open question regarding the forms of support they received from their employers, with the option to indicate multiple types of support. As participants could provide multiple answers, the frequencies of mentions exceed the total number of respondents. Three main areas of support emerged:

1. Personal attention (*n* = 478 codes), especially empathy through conversations (*n* = 235 codes) and gestures such as hugs and offers of help (*n* = 125 codes). Participants described support such as: *“Time off to visit the psychologist”*, *“I didn’t have to take half a day off to attend a funeral”*, and *“I was sent home with the clear statement that I should get a sick note”*.
2. Supportive attitudes (*n* = 403 codes), primarily “*understanding for depressed mood”* (3e Pos. 590) and compassion (*n* = 260 codes). Participants highlighted small but meaningful gestures, for example: *“Snacks for breaks, e.g., cake or sandwiches”* and *“Leaving the workplace on a day when grief overwhelmed me”*.
3. Structural adjustments (*n* = 320 codes). These included “*taking on work*”, “*being able to take time off without difficulty*” and individual work adjustments (*n* = 85 codes) such as “*the possibility of more breaks*” or “*flexible working hours*”. Some negative examples were also reported, reflecting a lack of support or insensitive behavior, including: *“My mistakes were not addressed”*, *“Hollow phrases”*, and *“My boss forced me to return to work two days after my husband’s death”*.

### Ways of support provided to colleagues

319 participants described a wide range of ways in which they had supported colleagues who were facing serious illness, dying, death or grief. The most frequently reported theme was personal offers of help (*n* = 593 codes), with conversations (*n* = 393 codes) and personal gestures (*n* = 91 codes) being the most common ways. Further subthemes included verbal encouragement (*n* = 44 codes), practical assistance with daily tasks (*n* = 39 codes), and distraction, such as joint activities after work (*n* = 26 codes).

Participants also reported attitudinal ways of support, particularly expressions of compassion (*n* = 88 codes) and comfort (*n* = 56 codes). More active stances, such as taking sides with affected colleagues in conflicts, were also mentioned.

Structural adjustments were another important form of support (*n* = 282 codes), including task redistribution or workload reduction (*n* = 150 codes), individual work adjustments (*n* = 58 codes), and leave arrangements (*n* = 34 codes). Additional responses referred to financial support (*n* = 23 codes) and professional assistance (*n* = 17 codes).

However, some participants reported barriers to providing support. These included organizational barriers (*n* = 74 codes), a self-perceived lack of competence (*n* = 54 codes), uncertainty about how to act (*n* = 27 codes), and the assumption that support was not desired by the affected individuals (*n* = 39 codes). *“The topic of death and my own mortality is extremely difficult for me to cope with, and I do not know how to react. I feel uncomfortable.”*

## Free-text responses: Target state of support regarding serious illness, dying, death, and grief in the workplace

### Emotional, Informational, and Structural Support

Overall, 702 participants wished for greater confidence in dealing with individuals affected by serious illness, dying, death, or grief, whereas 425 did not. The most frequently expressed need was for training and information on these topics (*n* = 242 codes): *“Perhaps a short course on how to deal with it. Perhaps a good brochure would also suffice”*. Within the framework of organizational culture, designated contact points were identified as a potential source of support (*n* = 48 codes): *“A defined contact person in (...) so that you don’t have to inform your superiors yourself when you are under this stress. A kind of confidant to whom you can communicate your fears/thoughts/concerns about the situation in combination with work (...) who takes over discussions with superiors”*. The desire for normality and openness regarding topics such as death and grief was also perceived as helpful (*n* = 38 codes), as was an attitude characterized by understanding and compassion (*n* = 33 codes), and the opportunity for discussion (*n* = 41 codes). Responses from managers and non-managers were relatively similar, suggesting comparable perceptions across hierarchical levels.

Accordingly, sympathy (*n* = 117 codes), consideration (*n* = 75 codes), and supportive conversations (*n* = 108 codes) were reported as beneficial following experiences with serious illness, dying, death or grief. *“A colleague who listened to me. She didn´t try to sugarcoat the situation; she was just there and listened.”*

Although structural adjustments, such as time off, were less frequently described as helpful (*n* = 43 codes), they were still perceived positively when provided (*n* = 126 codes). However, some participants reported not receiving leave (*n* = 36 codes) or lacking workplace accommodations (*n* = 39 codes). Negative experiences reinforce deficits and were associated with a lack of compassion (*n* = 117 codes), ignorance (*n* = 75 codes), and inappropriate comments (*n* = 43 codes): *“When I suffered another bereavement, a colleague who doesn’t particularly like me said, ‘Life goes on’”*.

### Feasible support measures in the workplace

Among the 285 managers with personnel responsibilities who completed the online survey, 95 reported possibilities for support in situations involving stress due to serious illness, dying, death, and grief. They identified the following structural measures as feasible options: leave of absence (*n* = 63 codes), flexible working hours (*n* = 38 codes), and access to professional contact persons (*n* = 19 codes). Similar wishes were expressed by non-managers, who also emphasized leave of absence (*n* = 140 codes of 521 respondents) and workplace adjustments (*n* = 91 codes). One participant stated: “*A one-to-one conversation about the extent of work I am able to manage”*. While professional contact persons were rarely mentioned by non-managers, as their responses mainly referred to leadership behaviors.

By contrast, considerably fewer managers with personnel responsibility referred to attitudinal aspects such as empathy and compassion (*n* = 9 codes) or consideration and understanding (*n* = 14 codes). However, these were strongly desired by affected employees (consideration and understanding; *n* = 361 codes). Several managers indicated that they saw little or no possibility for supportive measures (n = 64 codes), arguing that such experiences were *“a private matter and unrelated to the workplace”*.

### Desired support from a grief contact person

In response to the question of what employees would wish for in a potential contact person for dealing with serious illness, dying, death, and grief, both managers (*n* = 130 codes of 296 respondents) and non-managers (*n* = 169 codes of 533 respondents) highlighted attitudinal qualities, foremost understanding and compassion. Conversations were considered a central form of support (managers: *n* = 39 codes; non-managers: *n* = 134 codes), helping employees *“reintegrate into normal work life, which for many represents their livelihood”*. Availability was also considered crucial (managers: *n* = 22 codes; non-managers: *n* = 80 codes): *“They should be easily accessible and not under time pressure”*. Professional competencies, such as training or experience, were mentioned less frequently (managers: *n* = 35 codes; non-managers: *n* = 53 codes), suggesting that *“the highest social competence”* is more essential. Some participants noted that “*just knowing that this person exists would ease the work climate in an extreme situation”*. However, only a few saw no need for such a contact (managers: *n* = 14 codes; non-managers: *n* = 12 codes), arguing that supervisors should assume this role: *“I do not find a dedicated person appropriate; I would like to take on that role as a supervisor”*.

## Discussion

### Summary of results

The majority of employers and employees have been personally or indirectly affected by serious illness, dying, death or grief, with substantial impacts on their work and emotional well-being. This is in line with other findings where 96% of working-age employees had at least one bereavement in their working life (i.e., loss by death) [5].

Company size explained only a small proportion of the variance, but employees in small and medium-sized enterprises more often reported concerns about legal consequences and expressed a stronger desire for both emotional and structural workplace support. In contrast, larger companies were more likely to offer formalized guidelines, programs, and contact points.

Formal organizational resources for coping with serious illness, dying, death, and grief are limited, particularly in the psychosocial and educational domains. In contrast, informal interpersonal support from colleagues, co-workers, and managers is more common and is often perceived as more valuable than formal structural provisions. Although support is frequently offered, employees report barriers and uncertainties, highlighting a clear need for guidance and training. Importantly, employees tend to value attitudinal and relational aspects, such as understanding, empathy, and availability, whereas managers often emphasize structural measures, such as leave or workplace adjustments. This indicates a mismatch between employees’ needs and the managerial focus.

## Gaps and Opportunities in Workplace Bereavement Policies

### Legal and Organizational Context

Our findings highlight substantial gaps in organizational bereavement support. Although the UK has often been considered a forerunner in establishing formal bereavement policies, even there, 27% of employees reported that their employer had no such policy, and 31% were unsure [22]. In our study, 50% of Germans stated that their organization offered no initiatives or programs in the event of bereavement, and 25% were uncertain.

Although bereavement leave is legally recognized in Germany, it is not universally available or standardized, and similar provisions are uncommon in other countries, such as the US. This underlines the need for highly individualized support opportunities rather than one-size-fits-all approaches [23].

Our results underscore that employees value empathy, understanding, and compassionate communication, which aligns with previous findings [22,24]. Notably, support does not necessarily have to take the form of standardized Employee Assistance Programs (EAPs). Such programs, as seen in the UK or Canada, provide counseling and consultation services to help employees and their families prevent or resolve personal difficulties [25]. However, support can also include more customizable measures, such as temporary workload adjustments [23].

Recent guidance from the Chartered Institute of Personnel and Development [26], which serves as an annual benchmark of good work and job quality in the UK, emphasizes the importance of comprehensive bereavement policies. Such policies should empower managers, provide access to workplace support services such as counseling, and offer clear information on leave entitlements. Our findings suggest that such guidance is highly relevant for German workplaces, which appear to face comparable challenges in balancing structural and psychosocial support.

### Needs and preferences regarding grief support

Despite these recommendations, gaps in training remain a persistent challenge [24], a finding that is mirrored in our study, where employees expressed a clear wish for information and training on dealing with issues of serious illness, dying, death, and grief. Dewhorst and colleagues [24] further suggest that bereavement training may be key to ensure that managers are adequately prepared to support grieving employees.

Our study complements the C.A.R.E. model of employee bereavement support [16], which identifies four critical components of effective support: Communication, Accommodation, Recognition of the loss, and Emotional support. The model provides a practical framework for organizations to operationalize grief support and aligns well with our findings that employees value empathy, understanding, and structural accommodations. Similar to Gilbert et al. (2021), our results indicate that effective support requires a combination of structural and psychosocial adjustments. Implementing such measures can promote grief literacy within workplaces, fostering compassionate organizational cultures [24,27]. Furthermore, evidence suggests that employees perceive the mere availability of supportive offerings positively; those who make use of available programs report higher levels of perceived support [23]. Particularly important were resources that facilitated communication and concrete workload adjustments. This highlights the need for workplaces to reduce barriers to uptake, normalize conversations about grief, and actively encourage the use of supportive measures [23].

Similarly, caregiving responsibilities can substantially affect employee performance and attendance. About 46% of employed caregivers report reduced work performance, and around 40% miss work or leave suddenly due to caregiving [28], with full-time caregivers also more likely to experience sickness-related absenteeism [29]. These findings highlight the need for workplaces to provide supportive environments that mitigate negative impacts on both performance and well-being.

Overall, these findings underscore the importance of integrating both flexible structural arrangements and empathetic psychosocial support into organizational practices in order to adequately meet the needs of employees experiencing grief.

### Remote versus workplace settings

The shift towards remote working presents both opportunities and challenges for grief support. While some participants appreciate the privacy and flexibility of working from home, others experience a sense of loss due to the absence of collegial support and social connectedness [22]. This suggests that a hybrid approach combining flexible working with intentional opportunities for psychosocial support may be needed.

### Strengths and limitations

A notable strength of this study is its large, nationally representative sample which reflects the distribution of sex, age groups, and federal states within the German adult population. The robustness of the study is further enhanced by the comprehensive assessment of experiences in the workplace regarding severe illness, dying, death, and grief, which was conducted through a detailed, multi-question survey. However, the cross-sectional design does not allow for causal inferences, and the findings reflect self-reported perceptions, which may be affected by recall or social desirability bias. Nevertheless, the breadth of perspectives provides valuable insights into an under-researched area.

One limitation of this study is the use of a self-developed, non-standardized questionnaire. While this enabled the survey to be tailored to the aims of the study, the lack of established psychometric properties may limit its generalizability and comparability with other studies.

Of the 15,203 individuals invited, 15% initiated the survey and 47% of these completed it, corresponding to 7% of all invitees. Although completion among starters was moderate, the overall participation relative to invitations may limit representativeness and should be considered when interpreting the findings. Generalizability beyond the study sample should therefore be approached with caution.

The inclusion of open-ended questions was a notable strength. It enabled participants to provide rich, detailed insights into their experiences and perspectives. The study achieved a large nationwide sample with quota-based representation across age groups and federal states in Germany. However, the use of a non-probability online panel and snowball sampling may have introduced self-selection bias, as participants who chose to take part may differ systematically from the broader working population [30]. Online methods may exclude individuals with limited digital access or skills. However, compared with face-to-face interviews, this method may encourage participants to share more openly about sensitive or personal matters [31].

## Conclusion

This study reveals a clear gap between employees’ needs and managerial perspectives on serious illness, dying, death, and grief in the workplace. While managers mainly stress structural solutions such as leave and workload adjustments, employees strongly emphasize the importance of empathy, understanding, and open dialogue.

A key finding is the lack of grief-related training despite widespread recognition of its importance. Promoting literacy around grief and serious illness among leaders and staff emerges as a key step towards creating more compassionate workplaces. Importantly, organizational measures are perceived as supportive not only when available but when actively used, underscoring the value of encouraging uptake.

While flexible work arrangements and digital solutions can provide support, they can also lead to social isolation, highlighting the need for tailored approaches. Overall, workplaces have the potential to function as compassionate communities. By integrating structural provisions with interpersonal sensitivity, they can enhance both employee well-being and organizational resilience.
